# Noninfectious Retrovirus Particles Drive the *Apobec3*/*Rfv3* Dependent Neutralizing Antibody Response

**DOI:** 10.1371/journal.ppat.1002284

**Published:** 2011-10-06

**Authors:** Diana S. Smith, Kejun Guo, Bradley S. Barrett, Karl J. Heilman, Leonard H. Evans, Kim J. Hasenkrug, Warner C. Greene, Mario L. Santiago

**Affiliations:** 1 Department of Medicine, University of Colorado Denver, Aurora, Colorado, United States of America; 2 Rocky Mountain Laboratories, National Institutes of Allergy and Infectious Diseases, Hamilton, Montana, United States of America; 3 Gladstone Institute of Virology and Immunology, University of California San Francisco, San Francisco, California, United States of America; 4 Departments of Medicine, Microbiology and Immunology, University of California San Francisco, San Francisco, California, United States of America; 5 Department of Immunology, University of Colorado Denver, Aurora, Colorado, United States of America; 6 Department of Microbiology, University of Colorado Denver, Aurora, Colorado, United States of America; Fred Hutchinson Cancer Research Center, United States of America

## Abstract

Members of the *APOBEC3* family of deoxycytidine deaminases counteract a broad range of retroviruses *in vitro* through an indirect mechanism that requires virion incorporation and inhibition of reverse transcription and/or hypermutation of minus strand transcripts in the next target cell. The selective advantage to the host of this indirect restriction mechanism remains unclear, but valuable insights may be gained by studying APOBEC3 function *in vivo*. *Apobec3* was previously shown to encode *Rfv3*, a classical resistance gene that controls the recovery of mice from pathogenic Friend retrovirus (FV) infection by promoting a more potent neutralizing antibody (NAb) response. The underlying mechanism does not involve a direct effect of Apobec3 on B cell function. Here we show that while Apobec3 decreased titers of infectious virus during acute FV infection, plasma viral RNA loads were maintained, indicating substantial release of noninfectious particles *in vivo*. The lack of plasma virion infectivity was associated with a significant post-entry block during early reverse transcription rather than G-to-A hypermutation. The Apobec3-dependent NAb response correlated with IgG binding titers against native, but not detergent-lysed virions. These findings indicate that innate Apobec3 restriction promotes NAb responses by maintaining high concentrations of virions with native B cell epitopes, but in the context of low virion infectivity. Finally, Apobec3 restriction was found to be saturable *in vivo*, since increasing FV inoculum doses resulted in decreased Apobec3 inhibition. By analogy, maximizing the release of noninfectious particles by modulating *APOBEC3* expression may improve humoral immunity against pathogenic human retroviral infections.

## Author Summary

Members of the *APOBEC3* gene family can potently inhibit a broad range of retroviruses, including HIV-1. In cell culture, APOBEC3 counteracts retroviruses by: (1) reducing the infectivity of virions; and (2) inducing lethal G-to-A hypermutation in the next target cell. The selective advantage to the host of an ‘indirect’ restriction factor that is incorporated into virions and acts in the next target cell remains mysterious. We previously showed that *Apobec3* encodes *Rfv3*, a classical resistance gene that controls the neutralizing antibody response against Friend retrovirus infection in mice. Here we demonstrate that Apobec3 promotes the release of substantial levels of noninfectious virions in the plasma during acute FV infection, resulting in a more potent antibody response directed against intact virions. Thus, we propose that APOBEC3 evolved as an innate mechanism to promote high concentrations of retrovirus antigen in a native but noninfectious form to effectively prime the neutralizing antibody response. These findings could have important implications for improving HIV-1 specific antibody responses.

## Introduction

Millions of years of co-evolution of retroviruses and mammalian hosts has led to the emergence of retroviral host restriction factors, some of which were discovered following major efforts to understand key steps in the HIV life cycle. Most of these genes, such as TRIM5α [1], Tetherin/Bst2 [2] and SAMHD1 [3], restrict retroviruses in the infected cell. In contrast, members of the *APOBEC3* family of deoxycytidine deaminases are distinguished by their ability to inhibit retroviruses in the *next* target cell. In cell culture, co-transfection of *APOBEC3* expression plasmids with retrovirus molecular clones does not decrease virus output, but the infectivity of the resulting virions is dramatically decreased [4]–[5]. These APOBEC3-containing virions are fusion-competent, but encounter post-entry blocks from early reverse transcription to integration [6]–[7], with G-to-A hypermutation of nascent reverse transcripts observed in most, but not all [8]–[11] retrovirus infections. Notably, while *in vivo* studies have largely focused on G-to-A hypermutation as a read-out of APOBEC3 function [12]–[14], the biological relevance of APOBEC3-mediated reduction of virion infectivity remains unclear. In fact, it is currently unknown whether high viral output with reduced infectivity can be detected *in vivo*, since multiple rounds of replication with Apobec3 restriction will ultimately result in decreased total virus titers [15]–[16].

Investigating the impact of APOBEC3 restriction *in vivo* is logistically difficult in humans due to potential redundancy in antiretroviral activities of seven human APOBEC3 members (APOBEC3A, B, C, D, F, G and H) (reviewed in [17]). Moreover, APOBEC3 activity is likely most relevant immediately following viral transmission, but such biological samples are very difficult to obtain from pathogenic human retrovirus infections. In contrast, mice encode a single *Apobec3* gene (*mA3*) [4]–[5] that could be genetically disrupted. While *mA3*-deficient mice are physiologically normal [18], they proved more susceptible to pathogenic murine retroviral infections that include Mouse Mammary Tumor virus [8], Moloney Murine Leukemia Virus [19] and Friend retrovirus (FV) complex [20]–[21]. These studies provide a springboard for investigating the immunological impact of mA3 restriction *in vivo*.

FV causes severe splenomegaly and erythroleukemia in adult immunocompetent mice, and resistance and susceptibility to FV have been mapped to a variety of genes [22]–[23]. One of these genes, *Recovery from Friend retrovirus gene 3 (Rfv3)*, influences the recovery of mice from viremia by promoting a more potent neutralizing antibody (NAb) response [24]–[25]. C57BL/6 (B6) mice recover from viremia, develop stronger NAb responses and are *Rfv3* resistant, while BALB/c, A.BY and A/WySn strains have persistent viremia, develop weaker NAb responses and are *Rfv3* susceptible. Our group and others demonstrated that the B6 *mA3* gene acts as the classical *Rfv3* resistance gene, promoting stronger NAb responses and facilitating recovery from FV viremia, infection, and disease in (B6×BALB/c)F_1_, (B6×A.BY)F_1_ and (B6×A/WySn)F_1_ mice [20], [26]–[27]. In addition, the B6 *mA3* gene restricted acute FV replication in immune compartments [20]–[21], [27]–[28]. Acute FV inhibition was associated with significantly higher *mA3* mRNA expression and splicing differences in *Rfv3* resistant (B6) compared to *Rfv3* susceptible strains (BALB/c, A.BY, A/WySn) [10], [21], [26], [29]. However, the mechanism through which B6 *mA3* promotes FV-specific NAb responses remains unknown.

The APOBEC3 genes are evolutionarily related to Activation-Induced Deaminase, a B-cell specific enzyme that is critical for antibody affinity maturation and class-switching [30]. Thus, the identification of *mA3* as *Rfv3* led to the immediate hypothesis that *mA3* may directly influence antibody development [20]. However, hapten immunization studies revealed that B6 *mA3* influenced antibody affinity maturation only in the context of FV infection [28]. Thus, the underlying mechanism for the *mA3*/*Rfv3* phenotype does not involve a direct effect of mA3 on B cell function. In fact, decreased immune dysfunction was found to be a critical component of how B6 *mA3* promotes NAb responses [27]–[28]. We therefore hypothesized that mA3 influences NAb responses by promoting the release of noninfectious virions *in vivo* [28], driving NAb responses without eliciting pathology.

The mechanism for the *mA3*/*Rfv3* phenotype may have implications for improving humoral immunity against human retroviruses, particularly against HIV-1. However, HIV-1 encodes Vif, which promotes the degradation of the human homologues APOBEC3G (hA3G) and APOBEC3F (hA3F) [31]–[34]. Surprisingly, despite the action of Vif, hA3G/hA3F-mediated G-to-A hypermutation was detected in HIV-1 sequences from clinical specimens [12]–[14]. These findings could in part be due to the emergence of defective Vif alleles [35]. However, high *hA3G* mRNA levels in primary cells or tissues were also associated with lower HIV-1 viral loads [36]–[37]. In rhesus macaques infected with SIV, rhesus macaque *A3G* (*rhA3G*) levels in colonic biopsies following mucosal vaccination also correlated with set-point plasma viral loads [38]. Thus, hA3G/hA3F may be induced to levels that saturate endogenous levels of Vif. This is consistent with *in vitro* observations that show inhibition of wild-type HIV-1 with increasing *hA3G* transfection levels [4]–[5]. Unfortunately, obtaining direct evidence that innate hA3G/hA3F restriction is saturable in humans is not feasible.

We therefore evaluated the *in vivo* antiviral activity and saturability of B6 *mA3* in the context of FV infection of mice. The results provide long-sought insights into a fundamental APOBEC3 restriction phenotype that may have important implications for HIV-1 vaccine research.

## Results

### B6 *mA3* promotes substantial release of noninfectious retroviral particles *in vivo*

**Figure 1:**
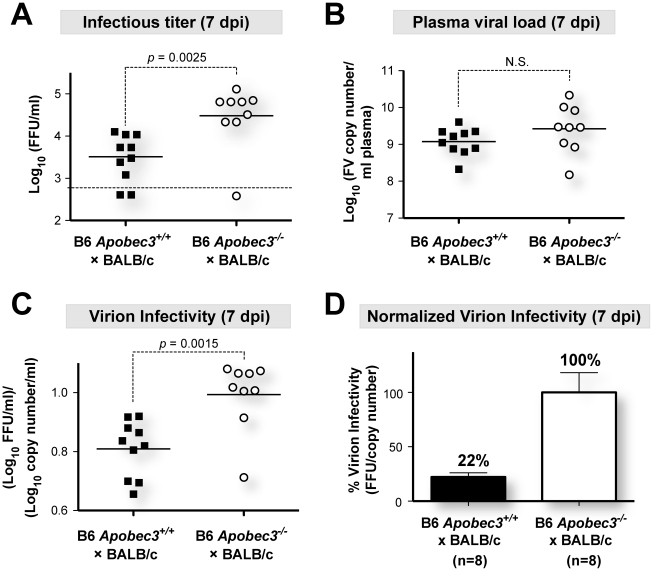
B6 *mA3* promotes noninfectious particle release during acute FV infection. (B6×BALB/c)F_1_ mice were infected with 140 SFFU of FV complex and 7 dpi plasma samples from B6 *mA3* ^+^ and B6 *mA3* ^−^ F_1_ mice were subjected to infectious viremia titration in *Mus dunni* cells and viral RNA copy determinations by quantitative PCR. Virion infectivity for each sample was measured by taking the ratio of infectious titer and viral load. (A) Infectious viremia and (B) plasma viral load during acute infection of (B6×BALB/c)F_1_ mice are shown as log_10_ values. (C) Virion infectivity was calculated by taking the ratio of log_10_ infectious titer and plasma viral load. (D) Virion infectivity was also compared with non-log transformed values, setting the average infectious titer per viral copy number of (B6 *mA3* ^−/−^×BALB/c)F_1_ as 100%. Samples below the assay limit of detection (below the dotted lines in panel A) were excluded in this calculation. Solid lines correspond to mean values, and *p* values from a two-tailed Student's *t* test are shown. Each dot corresponds to an infected mouse. Error bars correspond to the standard error of the mean.

(B6×BALB/c)F_1_ mice (genotype *Rfv3* ^r/s^) are highly susceptible to FV infection but recover from plasma viremia by 28 days post-infection (dpi) due to the dominant, B6-encoded *Rfv3* resistance gene [20], [26] (Figure S1A in Text S1). In contrast, the majority of (B6 *mA3* ^−/−^×BALB/c)F_1_ mice (genotype *Rfv3* ^−/s^) do not survive to 28 dpi, and those that survive display elevated plasma viremia, consistent with identity between *Rfv3* and *mA3* [20], [26] (Figure S1B in Text S1). We previously reported that the significant survival disadvantage of (B6 *mA3* ^−/−^×BALB/c)F_1_ mice was linked to higher plasma viremia during acute infection (7 dpi), quantified on susceptible *Mus dunni* cells using an FV envelope-specific monoclonal antibody to detect foci of infectivity [39]. Thus, the *Mus dunni* assay measures infectious viremia. In contrast, a quantitative RT-PCR assay measuring total viral RNA copies (specifically, the F-MuLV helper virus component, as described in Materials and Methods) does not distinguish between infectious and noninfectious virions [26], [40]. To determine if B6 *mA3* affected the relative amount of infectious virions released *in vivo*, we measured the ratio of virus titers obtained from both assays in plasma from (B6×BALB/c)F_1_ mice infected with 140 SFFU of FV (Figure 1).

Interestingly, plasma samples from (B6 *mA3^+/+^*×BALB/c)F_1_ mice that had 10-fold lower infectious viremia titers at 7 dpi compared to (B6 *mA3* ^−/−^×BALB/c)F_1_ mice (Figure 1A) had equivalent total viral RNA loads (Figure 1B). Thus, the fraction of infectious viral particles was significantly higher in mice without a functional B6 *mA3* gene (Figure 1C). By setting the mean virion infectivity of (B6 *mA3* ^−/−^×BALB/c)F_1_ mice to 100%, the relative infectivity of plasma virions from (B6 *mA3^+/+^*×BALB/c)F_1_ mice averaged only 22% (Figure 1D). Thus, B6 *mA3* restriction resulted in approximately 5-fold higher levels of noninfectious virions at 7 dpi. Higher proportions of noninfectious particles were also observed in (B6 *mA3* ^+/+^×A.BY)F_1_ versus (B6 *mA3* ^−/−^×A.BY)F_1_ mice (Figure S2 in Text S1).

### Minimal mA3-associated G-to-A substitutions in viral RNA and reverse transcripts from plasma virions isolated during acute infection

**Figure 2:**
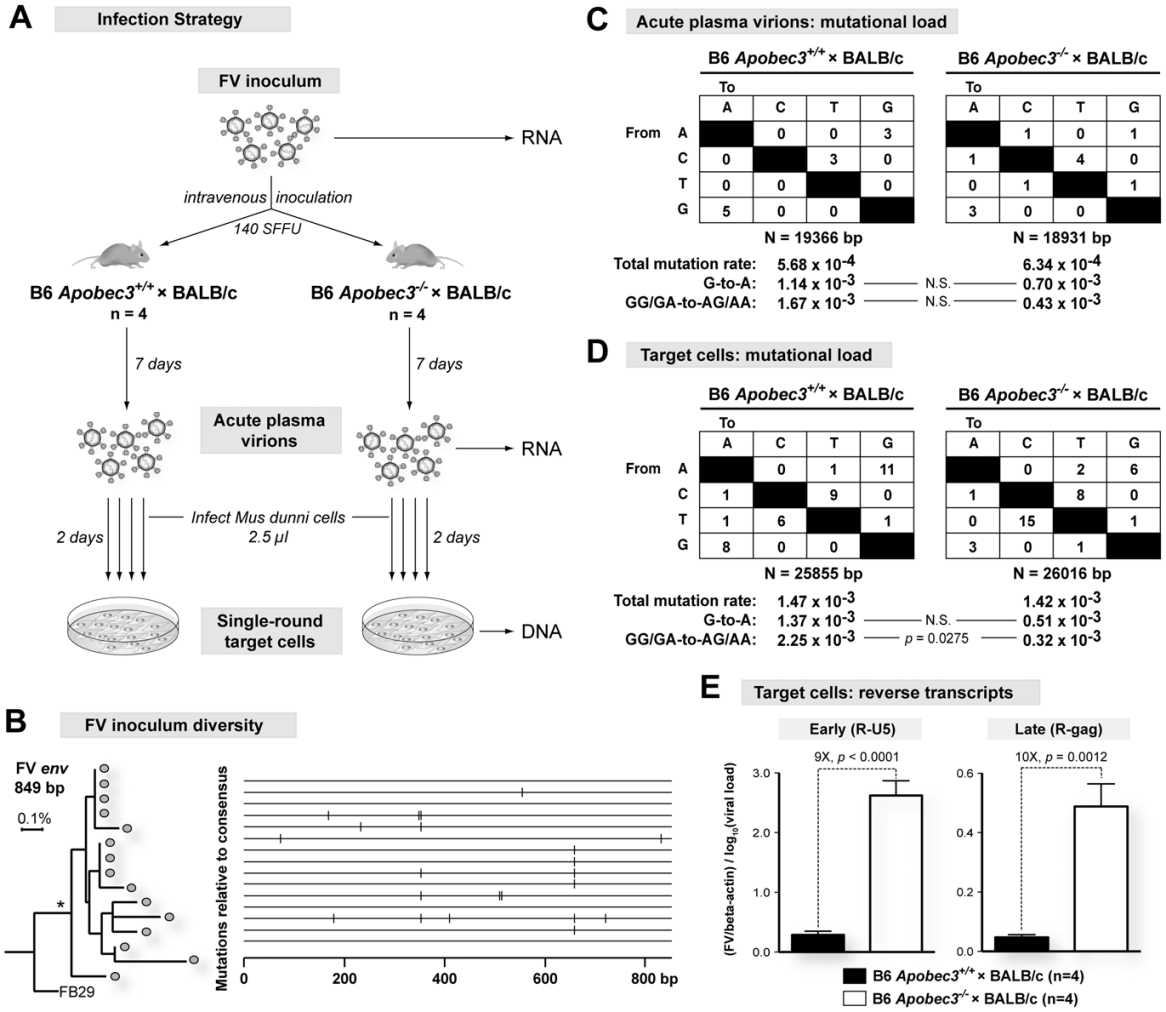
Mechanism of B6 *mA3* inhibition of plasma virion infectivity. (A) General strategy to investigate FV evolution *in vivo*. FV *env* sequences from the inoculum stock, 7 dpi plasma, and reverse transcripts following infection of target *Mus dunni* cells with 7 dpi plasma were compared with each other. (B) Sequence characterization of the FV inoculum quasispecies. (*Left*) Contemporary FV sequences (gray circles) cluster in phylogenetic analyses with >80% bootstrap value (asterisk), and diverge from a 1983 sequence, FB29. (*Right*) Sequence alignment against the consensus showed 13 variant sites (vertical bars). These variations were excluded from subsequent analyses of FV mutation rates in infected mice. The two most divergent FV sequences differed by 0.6% in nucleotide identity. (C) Virion RNA mutational loads from (B6×BALB/c)F_1_ mice (140 SFFU) 7 dpi plasma were compared against the FV inoculum consensus. No significant difference in mutation rates was observed (Chi-Square test). (D) Reverse transcripts following infection of *Mus dunni* cells with plasma virions were amplified using conditions to bias for G-to-A mutational detection. Substitution rates were tabulated from a combined consensus sequence derived from the FV inoculum (panel B) and the corresponding plasma virions (panel C). mA3-associated G-to-A substitutions is associated with (B6 *mA3* ^+/+^×BALB/c)F_1_ status by Chi-Square test. (E) mA3 inhibits FV early reverse transcription. DNA from *Mus dunni* cells infected for 2 days with plasma virions were subjected to quantitative PCR for early (R-U5) and late (R-gag) reverse transcripts, and normalized to beta-actin copy number and input virus. Differences in means were analyzed using a two-tailed Student's *t*-test.

In the absence of HIV-1 Vif, hA3G and hA3F mediate high levels of G-to-A hypermutation in the minus strand of viral DNA, disrupting open reading frames and effectively inactivating HIV-1. In contrast, mA3 did not appear to induce G-to-A hypermutation against FV, MMTV and MLV [8]–[11], but these data were obtained either from cells infected *in vitro* or from bulk infected tissues. To investigate the mechanism of B6 *mA3* restriction of plasma virions released during acute infection *in vivo*, we monitored FV sequence evolution in plasma from infected (B6 *mA3* ^+/+^×BALB/c)F_1_ and (B6 *mA3* ^−/−^×BALB/c)F_1_ mice (Figure 2A). Partial *env* sequences amplified from the FV inoculum stock phylogenetically clustered with each other (Fig. 2B, *left panel*), supporting their authenticity as reference sequences. Mutations that were already present in the inoculum quasispecies (Figure 2B, *right panel*) were then excluded from FV mutational analyses in infected mice.

Multiple FV *env* sequences were obtained from 7 dpi plasma of (B6 *mA3* ^+/+^×BALB/c)F_1_ and (B6 *mA3* ^−/−^×BALB/c)F_1_ mice. Relative to the FV inoculum sequences, the cumulative mutational load (Figure 2C), G-to-A substitutions (Figure 2C), and the continuity of the envelope open reading frames (Figure S3 in Text S1) in plasma viral RNA from (B6 *mA3* ^+/+^×BALB/c)F_1_ and (B6 *mA3* ^−/−^×BALB/c)F_1_ mice did not significantly differ from each other. Thus, the reduction in the infectivity of 7 dpi plasma virions from (B6 *mA3* ^+/+^×BALB/c)F_1_ mice (Figure 1C) was likely not due to disproportionately mutated viral genomes.

Newly formed FV reverse transcripts in target cells following infection with plasma virions were next evaluated using approaches to bias for detection of G-to-A mutations [11], [41]–[42]. When compared to FV sequences from the inoculum and the corresponding 7 dpi plasma samples, similar mutation frequencies and total G-to-A substitutions were observed in (B6 *mA3* ^+/+^×BALB/c)F_1_ and (B6 *mA3* ^−/−^×BALB/c)F_1_ mice (Figure 2D). However, when these G-to-A substitutions were partitioned into mA3-associated dinucleotide preferences, the (B6 *mA3^+/+^*×BALB/c)F_1_ strain was associated with the detection of GG→AG and GA→AA mutations (Figure 2D). Thus, we could detect signatures of mA3-associated G-to-A mutations from reverse transcripts generated from acute B6 *mA3* ^+^ plasma virions. However, even with techniques that significantly biased for the detection of such reverse transcripts, the G-to-A substitution frequency obtained for FV (0.14%) was at least 10-fold lower than that observed for HIV-1 ΔVif, which ranges from 1.3 to 6.5% [14]. Together, these findings suggest that mA3-mediated deamination plays a very minor role in restricting FV infection *in vivo*.

### Acute plasma virions from (B6 *mA3* ^+/+^×BALB/c)F_1_ mice are inhibited at the earliest stages of reverse transcription

The lack of mA3-associated G-to-A substitutions in reverse transcripts from acute plasma virions of (B6 *mA3* ^+/+^×BALB/c)F_1_ mice argued in favor of a deamination-independent mechanism of mA3 inhibition. Human A3G can non-enzymatically impair an early step in HIV-1 reverse transcription involving the generation of strong stop DNA [7], [43]. Accordingly, we quantified the levels of newly formed early (R-U5 or strong-stop DNA) and late (R-gag) reverse transcripts following single-round infection of target cells with 7 dpi plasma FV virions (Figure 2A). These studies revealed a 9-fold decrease in early reverse transcripts from (B6 *mA3* ^+/+^×BALB/c)F_1_ plasma virions compared to F_1_ mice lacking B6 *mA3* (Figure 2E, *left panel*). No further decrease was observed in late reverse transcripts (Figure 2E, *right panel*). Thus, the post-entry block conferred by B6 *mA3* on FV plasma virions occurred primarily during the earliest stages of reverse transcription.

### Decreased cellular FV infection levels in (B6 *mA3* ^+/+^×BALB/c)F_1_ mice

We previously reported that B6 *mA3* decreased FV infection levels in target cells that include erythroblasts and B cells in (B6×A.BY)F_1_ mice at 7 dpi [28]. FV infected cells were quantified by flow cytometry using a Glyco-Gag specific monoclonal antibody, MAb 34 [44]. We now extend this observation to (B6×BALB/c)F_1_ mice. As shown in Table 1, the percentage of MAb 34^+^ cells was consistently lower in (B6 *mA3^+/+^*×BALB/c)F_1_ compared to (B6 *mA3* ^−/−^×BALB/c)F_1_ strains in multiple cell subpopulations in the bone marrow and the spleen. Statistical significance was achieved with bone marrow erythroblasts and splenic B, T and dendritic cells. These findings revealed that at 7 dpi, B6 *mA3* restriction of FV virion infectivity coincided with decreased FV infection of multiple target cells *in vivo*.

**Table 1:** Cellular FV infection of (B6×BALB/c)F_1_ mice.

		Cohort^a^		
Cell subpopulation^a^	Marker	WT^b^	KO^b^	*p*-value^c^
Bone marrow				
Erythroid	Ter119^+^	19.20±3.16	32.44±4.48	0.0266
B cells	CD19^+^	20.43±3.52	30.95±4.32	0.0758
T cells	CD3^+^	23.40±3.39	32.48±4.56	0.1261
Dendritic cells	CD11c^+^	31.13±4.57	41.84±4.80	0.1273
Spleen^d^				
Erythroid	Ter119^+^	19.06±2.93	24.20±2.34	0.1972
B cells	CD19^+^	7.73±0.99	10.87±1.07	0.0474
T cells	CD3^+^	4.92±0.61	6.90±0.51	0.0259
Dendritic cells	CD11c^+^	26.80±3.84	40.39±3.40	0.0192

a Mice were infected with 140 SFFU of FV complex and samples collected at 7 dpi. Entries correspond to mean FV^+^ cells ± standard error, quantified using a Glyco-Gag specific antibody, MAb 34.

b WT (wild-type) corresponds to (B6 *mA3^+/+^*×BALB/c)F_1_ mice; KO (knockout) corresponds to (B6 *mA3^−/−^*×BALB/c)F_1_ mice. Samples sizes: WT, n = 9; KO, n = 8.

c *p*-values were computed using a two-tailed Student's *t* test.

d No significant difference in spleen mass was observed (Mean: WT = 172.2±12.8 mg; KO = 200.0±11.6 mg).

### B6 *mA3* is a saturable innate restriction factor *in vivo*

**Figure 3:**
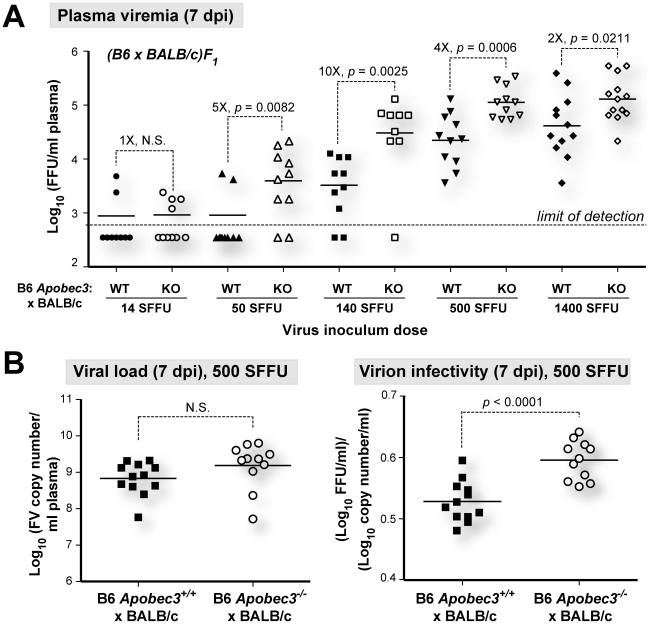
B6 *mA3* is a saturable innate restriction factor *in vivo*. (A) Saturability of innate mA3 restriction. Cohorts of (B6 *mA3^+/+^*×BALB/c)F_1_ and (B6 *mA3* ^−/−^×BALB/c)F_1_ mice were infected with varying doses of FV and infectious viremia in 7 dpi plasma samples were measured. The fold-difference in mean 7 dpi viremia between the wild-type and *mA3*-deficient F_1_ mice for each dose is shown. Dashed lines correspond to the limit of detection of the focal infectivity assay (600 FFU/ml). (B) Noninfectious FV release despite decreased *mA3* restriction. Plasma viral loads (*Left*) and virion infectivity (*right*) were determined for mice infected with 500 SFFU of FV. Plasma virion infectivity was significantly higher in (B6 *mA3^+/+^*×BALB/c)F_1_ compared to (B6 *mA3* ^−/−^×BALB/c)F_1_ mice. A similar result was observed with a lower infection dose (140 SFFU; Figure 1C). Each dot corresponds to an infected mouse. Differences in means were analyzed using a two-tailed Student's *t*-test.

To test whether B6 *mA3* restriction was saturable *in vivo*, we infected (B6 *mA3^+/+^*×BALB/c)F_1_ and (B6 *mA3* ^−/−^×BALB/c)F_1_ mice with titrated doses of FV, and measured infectious plasma viremia at 7 dpi (Figure 3A). (B6 *mA3* ^−/−^×BALB/c)F_1_ mice exhibited significantly higher 7 dpi infectious viremia compared to (B6 *mA3^+/+^*×BALB/c)F_1_ mice at all inoculum dosages, except at the lowest dose (14 SFFU), in which most titers were below the detection limit of the assay. The fold-difference in infectious viremia between (B6 *mA3^+/+^*×BALB/c)F_1_ and (B6 *mA3* ^−/−^×BALB/c)F_1_ cohorts varied with the dose of the viral inoculum. The fold-difference in B6 *mA3* restriction could not be accurately determined at 14 and 50 SFFU since several samples had infectious titers that were below the limit of detection. Maximum fold-difference in B6 *mA3*-mediated restriction was observed at 140 SFFU with 10-fold restriction (Figure 3A). At 500 SFFU and 1400 SFFU inoculum doses, there was only a 4-fold and 2-fold effect, respectively (Figure 3A). These results demonstrate that B6 *mA3*-mediated restriction was saturable *in vivo*. Notably, even at a higher viral dose that resulted in decreased B6 *mA3* inhibition, similar viral RNA loads (Figure 3B; *left panel*) and higher noninfectious particle release (Figure 3B; *right panel*) were still observed in (B6 *mA3^+/+^*×BALB/c)F_1_ versus (B6 *mA3* ^−/−^×BALB/c)F_1_ mice.

### The B6 *mA3*-dependent NAb response correlates with IgG titers against intact virions

**Figure 4:**
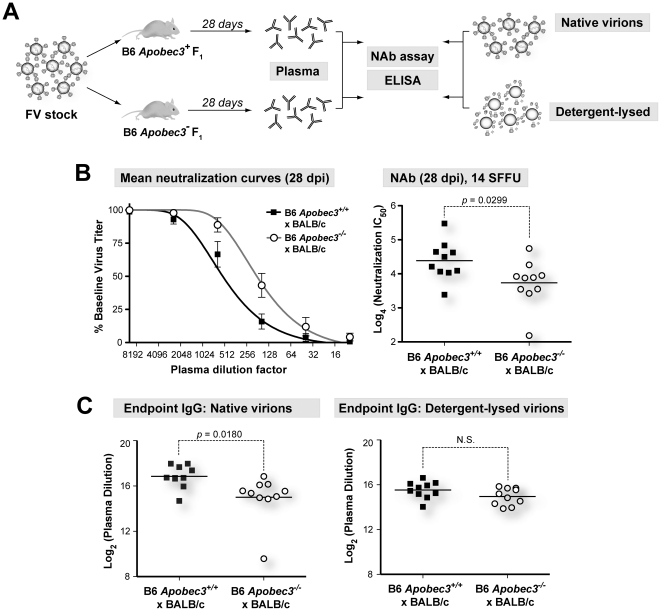
NAb responses in (B6×BALB/c)F_1_ mice. (A) Plasma samples at 28 dpi from (B6 *mA3^+/+^*×BALB/c)F_1_ and (B6 *mA3* ^−/−^×BALB/c)F_1_ mice were heat-inactivated and analyzed for neutralization potency and virion binding. (B) B6 *mA3* influences NAb responses in (B6×BALB/c)F_1_ mice. (*Left*) Neutralization curves were plotted (mean values are shown), and used to (*Right*) interpolate 50% inhibitory concentration (IC_50_) values. (C) B6 *mA3* dependent NAb responses correlate with IgG antibodies directed against native virions. Endpoint ELISAs were performed on individual plasma samples against virions that were not treated (native) or treated (detergent-lysed) with 1% Empigen-BB detergent. Values correspond to log_2_-transformed reciprocal plasma dilutions that corresponded to a cut-off based on 2× mean background absorbance. Each dot corresponds to an infected mouse. Differences in means were analyzed using a two-tailed Student's *t*-test.

The B6 *mA3* gene promoted NAb responses in (B6×A.BY)F_1_, (B6×A/WySn)F_1_ and pure B6 mice [20], [27]. However, measurements of NAb responses at 28 dpi in (B6×BALB/c)F_1_ mice were confounded by the increased mortality of (B6 *mA3* ^−/−^×BALB/c)F_1_ mice [20], [26]. In contrast, infection with 10-fold lower dose (14 SFFU) resulted in all of the mice surviving to 28 dpi, providing an opportunity to revisit this question in this F_1_ strain (Figure 4A). As shown in Figure 4B, (B6 *mA3^+/+^*×BALB/c)F_1_ mice developed significantly stronger NAb responses than (B6 *mA3* ^−/−^×BALB/c)F_1_ mice. Thus, the B6 *mA3* gene promoted FV-specific NAb responses in four genetic backgrounds that include (B6×BALB/c)F_1_, (B6×A.BY)F_1_, (B6×A/WySn)F_1_ and B6 mice.

To determine which components of the 28 dpi plasma correlated with B6 *mA3*-dependent neutralization, we evaluated the binding titers of the major immunologlobulin isotypes in plasma, IgM and IgG. Native virions were bound to 96-well plates, and endpoint IgM and IgG titers were determined by indirect ELISA. Endpoint IgM titers of 28 dpi plasma from B6 *mA3^+^* F_1_ and B6 *mA3* ^−^ F_1_ were not significantly different from each other (Figure S4 in Text S1). In contrast, endpoint IgG titers against native virions were significantly higher in (B6 *mA3^+/+^*×BALB/c)F_1_ compared to (B6 *mA3* ^−/−^×BALB/c)F_1_ mice (Figure 4C, *left panel*). Notably, this difference was not detected if detergent lysed-virions were used (Figure 4C, *right panel*). Similar results were observed for IgG endpoint titers in (B6 *mA3^+/+^*×A.BY)F_1_ versus (B6 *mA3* ^−/−^×A.BY)F_1_ mice (Figure S5 in Text S1). Thus, the B6 *mA3*-dependent antibody response was distinguished by an IgG response directed against intact virus particles.

## Discussion

### Mechanistic insights on the *mA3*/*Rfv3* phenotype

The molecular identification of the classical resistance gene *Rfv3* as *mA3* solved a 30-year mystery in retrovirology [20], [26]–[27]. However, this discovery unlocked new questions, foremost of which is the mechanism for how mA3 promotes NAb responses. Recent studies suggested an indirect mechanism that linked the ability of B6 *mA3* to restrict FV *in vivo* with a more vigorous B cell response [20]–[21], [27]–[28]. However, this finding seemed counterintuitive in light of studies on other viral infections, particularly HIV-1, which showed that viral antigen levels had to be preserved to maintain virus-specific antibody levels [45]–[47]. FV plasma viremia is routinely quantified using a focal infectivity assay that measures *infectious* virus [20], [39]. We therefore investigated whether this method underestimated the *total* numbers of FV particles in mice with functional Apobec3 activity.

**Figure 5:**
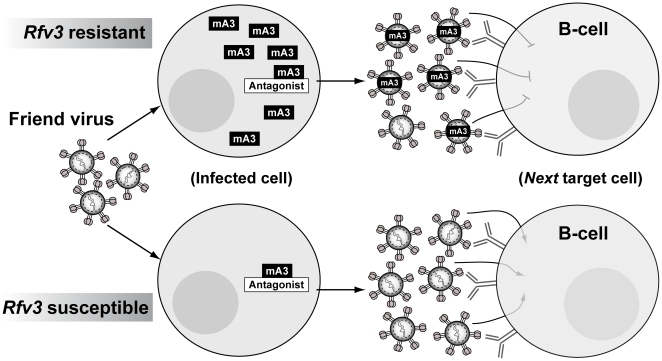
Model for mA3 action and FV-specific humoral immunity. In *Rfv3* resistant mice, high endogenous levels of mA3 may overwhelm putative FV-encoded antagonists such as Glyco-Gag, [57] resulting in functional mA3 incorporated into budding virus particles. In contrast, low levels of mA3 in *Rfv3* susceptible strains could efficiently be inactivated. In both strains, similar levels of virus particles released during acute infection could facilitate antigen-specific B cell development. However, virions derived from *Rfv3* resistant strains encounter an early post-entry reverse transcription block in target cells. Thus, antibody affinity maturation can occur against functional envelope trimers with decreased pathology in *Rfv3* resistant mice. In contrast, fully infectious virions from *Rfv3* susceptible strains could directly infect B cells (and other immune cells), resulting in immune dysfunction and weaker development of NAbs. We hypothesize a similar scenario during acute HIV-1 infection, except that: (1) the antagonist is HIV-1 Vif, which degrades hA3G/hA3F; and (2) the targets are CD4^+^ T cells, which in germinal centers are in direct contact with and provide help for antigen-specific B cell development.

Using a quantitative PCR assay for FV, B6 *mA3^+^* F_1_ mice exhibited plasma viral RNA loads that were similar to B6 *mA3*-deficient F_1_ mice at 7 dpi. In other words, B6 *mA3* activity led to no significant change in the physical numbers of virus particles. Instead, B6 *mA3* activity reduced *infectious* virus titers, indicating the release of substantial levels of noninfectious FV particles. These B6 *mA3*-restricted plasma virions, which account for up to 80% of virions released during acute infection relative to B6 *mA3* ^−^ F_1_ mice, encounter a significant post-entry block in early reverse transcription in the next target cell, resulting in reduced FV infection in multiple cellular targets *in vivo*, including splenic B cells. Thus, high levels of B6 *mA3*-restricted FV particles likely drove the FV-specific B cell response that resulted in the development of potent NAbs (Figure 5).

### APOBEC3-restricted virions as B-cell immunogens

Native virions are potent inducers of humoral immunity. Repeating molecular patterns on virions may be particularly effective in cross-linking and activating B cell receptors, while viral nucleic acids could enhance B cell responses by activating Toll-like receptors [48]. The FV envelope glycoprotein is the primary target of NAbs [49]–[50], and is organized as a trimeric spike in the native virion, analogous to the HIV-1 envelope glycoprotein [51]–[52]. Detergent treatment disrupts retroviral trimers into monomeric subunits. In this study, we show that the more potent NAb response from B6 *mA3^+^* F_1_ mice at 28 dpi correlated with significantly higher IgG binding titers against native, but not detergent-lysed virions. Thus, B6 *mA3*-restricted virions primed a more effective IgG response directed against native envelope trimers. Further studies are in progress to characterize the molecular attributes of this protective NAb response.

### Novel insights on a fundamental APOBEC3 restriction phenotype

APOBEC3 is unique among known virus restriction factors due the circuitous nature of its inhibitory mechanism. Instead of simply restricting retroviruses in the infected cell, APOBEC3 evolved the ability to incorporate into budding virions and restrict intact virions in the next target cell [4]–[5]. This biological property of APOBEC3 is conserved from rodents (mA3) to humans (hA3G), suggesting an important evolutionary advantage. However, despite nearly 10 years since the discovery of this fundamental APOBEC3 phenotype [4]–[5], the benefits of next-round inhibition by APOBEC3 to the host remain mysterious. Our findings suggest that mA3 restriction functions as an innate mechanism that allow B cell epitopes to be presented in the context of native virions, subsequently driving the NAb response. This humoral immune response is characterized by high specificity and memory, attributes that could allow the host to effectively control the infection as well as prevent subsequent infections.

### Implications for HIV-1 vaccine research

Although FV and HIV-1 infect different cell types and cause different diseases, functional similarities in APOBEC3 proteins from mice and humans suggest that concepts developed from the FV model may prove relevant to HIV-1 infection. Our current model on how mA3 promotes NAb responses implicates noninfectious virions as drivers of the Germinal Center (GC) cell response (Figure 5). These mA3 restricted virions retain immunogenicity but encounter a post-entry block in target cells that include B cells, reducing FV-induced immune dysfunction. While HIV-1 does not infect B cells, CD4^+^ T cells, the primary targets of HIV-1, also perform critical functions in the GC response, directly interacting with B cells to promote antigen-specific antibody development [53]. Thus, augmenting hA3G function during acute HIV-1 infection may preserve CD4^+^ T cell function in GCs and promote HIV-specific antibody development. In addition, the role of noninfectious virions in driving the *mA3*/*Rfv3* phenotype further support the use of native virion mimics such as virus-like particles or stabilized trimers as base scaffolds for vaccine design [54]–[55], with the caveat that more sophisticated approaches are needed to elicit NAbs that could broadly neutralize HIV-1 strains from multiple subtypes.

The existence of a lentiviral A3G antagonist, Vif, provides an opportunity to experimentally test whether modulating A3G function can improve the lentivirus-specific humoral immune response. In the SIV model, mutating the *Vif* gene in SIV to attenuate its function [56] may allow for rhA3G to promote non-infectious virion release and improve humoral immunity in infected rhesus macaques. Similar studies on HIV-1 infection in humans are not possible, but therapeutic agents that block the Vif-hA3G interaction could prove useful. Unfortunately, compounds independently confirmed to specifically inhibit the Vif-hA3G interaction have yet to be identified. In this study, we provide the first evidence that innate mA3 restriction is saturable *in vivo*, possibly reflecting a delicate balance between the endogenous levels of mA3 and a putative mA3 antagonist encoded by FV, Glyco-Gag [57]. Thus, inducing hA3G levels to saturate, rather than disrupt, the interaction with Vif may be a viable alternative to promote hA3G activity (Figure 5). Notably, Interferon-alpha (IFN-α, a cytokine that could induce APOBEC3G expression in HIV-1 target cells *in vitro* [58]–[60], improved the kinetics of HIV-1 specific antibody development when clinically administered during acute HIV-1 infection *in vivo* [61]. Further studies on the link between IFN-α and APOBEC3 *in vivo* may provide critical insights on whether the saturability of APOBEC3 restriction can be exploited for therapy and vaccine development.

## Materials and Methods

### Ethics statement

This study was carried out in strict accordance with the recommendations in the Guide for the Care and Use of Laboratory Animals of the National Institutes of Health. The protocol was approved by the University of Colorado Health Sciences Center Animal Care and Use Committee [Permit Number B-89709(10)1E]. All infections were performed under isoflurane anaesthesia, and all efforts were made to minimize suffering.

### Mice

B6, BALB/c and A.BY mice were purchased from The Jackson Laboratory. B6 *mA3* deficient mice were derived from the XN450 cell line [20] and backcrossed for 9 generations. Experimental groups consist primarily of (B6 *mA3^+/+^*×BALB/c)F_1_ versus (B6 *mA3^−/−^*×BALB/c)F_1_ mice. The rationale for an F_1_ transcomplementation approach is explained in more detail (Figure S1 in Text S1). Experiments were also performed in (B6 *mA3^+/+^*×A.BY)F_1_ versus (B6 *mA3^−/−^*×A.BY)F_1_ mice (Figures S2, S4 and S5 in Text S1). Note that B6, BALB/c and A.BY mice have a functional B cell Activating Factor Receptor (BAFF-R) and normal B cell maturation phenotype, as previously described [26]. These mice are also *Fv1* ^b^ and are therefore susceptible to B-tropic FV infection [62].

### Description of FV inoculum stock

Mice were infected with FV complex derived from *in vivo*-passaged stocks originally used to describe *Rfv3* [24], [63]. This B-tropic FV stock contains: replication-competent ecotropic Friend murine leukemia helper virus (F-MuLV); replication-defective spleen-focus forming virus (SFFV; Lilly-Steeves strain); lactate-dehydrogenase elevating virus (LDV). LDV is a ‘contaminant’ RNA virus that could enhance the pathogenicity of FV by delaying adaptive immune responses [64]–[65]. No polytropic or mink-cell focus-inducing viruses (MCFs) were detectable in the virus stock by focal immunofluorescence assay with antibodies Hy7 or mAb 516, which detect the vast majority of MCFs [66] (detection limit of 20/ml).

### FV infection

SFFV titers in the FV stock were titered in BALB/c mice and expressed as spleen focus forming units (SFFU) per ml. (B6×BALB/c)F_1_ mice were infected intravenously via the retro-orbital route with 14 to 1400 SFFU (spleen focus forming units) in 300 µl RPMI and (B6×A.BY)F_1_ mice were infected with 1400 SFFU. All mice were >2 months old. Plasma samples were harvested at 7 or 28 days post-infection (dpi).

### Infectious viremia titration

Infectious viremia titers were determined by serially diluting plasma into *Mus dunni* cells containing 4 µg/ml polybrene (Sigma; St Louis, MO). Infected cells were detected using a monoclonal antibody specific to F-MuLV gp70, MAb 720, as previously described [20], [39]. Briefly, F-MuLV gp70+ cells were detected following incubation with anti-mouse IgG conjugated to horseradish peroxidase and an insoluble substrate, 3-amino-9-ethylcarbazole (Sigma). Titers were expressed as log_10_ focus forming units (FFU) per ml of plasma.

### Plasma viral load

Viral RNA copy numbers were quantified by real-time PCR (qPCR) as described [26], [40]. RNA from plasma (10 µl) was extracted using the RNAeasy kit (Qiagen; Valencia, CA), and used as template for one-step reverse transcription and PCR (Applied Biosystems; Carlsbad, CA) using FV-specific primers (FLVsense: 5′-GGACAGAAACTACCGCCCTG and FLVantisense: 5′-ACAACCTCAGACAACGAAGTAAGA) and probe (FLVprobe: FAM-TCGCCACCCAGCAGTTTCAGCAGC-TAMRA). Copy numbers were interpolated from an in-plate T7-transcribed RNA standard, and expressed as log_10_ copy number per ml of plasma.

Sequence alignments of the F-MuLV helper virus (GenBank Accession #Z11128), the Lilly-Steeves SFFV strain (V01552.1) and MCFs (L. H. Evans, unpublished data) revealed that the FV qPCR primers have low to no significant identity with SFFV or MCFs (data not shown). Furthermore, we tested whether the FV qPCR primers could detect any endogenous polytropic *env* sequences present at one copy per mouse genome. The FV qPCR assay consistently detects an input of 100 copies of cloned F-MuLV DNA. If the qPCR primers cross-react with endogenous MLV, then we should obtain a positive signal if >100 copies of uninfected genomic DNA are added into the reaction. An input of 100 ng genomic DNA (∼34,000 genomes) from uninfected B6, A.BY and BALB/c mice into the qPCR reaction yielded no detectable signals. In contrast, >10^5^ copies were detected from 100 ng genomic DNA from FV infected B6 mice at 7 dpi (S. X. Li and M. L. Santiago, unpublished). Thus, we conclude that the qPCR assay is specific for the F-MuLV helper virus.

### Sequence analysis of FV inoculum and plasma viral RNA

RNA was extracted from the FV inoculum and acute plasma using the RNAEasy kit (Qiagen) and reverse-transcribed using the RT^2^ cDNA synthesis kit (SA Biosciences; Frederick, MD). FV *env* sequences (849 bp) were obtained by amplifying with primers FV.f (ACTTATTCCAACCATACCTCT) and FV.r (TTTAGCTGGTGGTATTGTTGA) using the Phusion Hi-Fidelity PCR kit (Finnzymes; Woburn, MA). Amplicons were cloned using the TOPO cloning kit (Invitrogen; Carlsbad, CA). FV inoculum sequences were aligned with FB29 and PVC-211 (GenBank Z11128 and M93134) using ClustalX (http://www.clustal.org/). Phylogenetic trees were constructed using the neighbor joining method with 1000 subreplicates. PVC-111 was used as outgroup. Viral RNA sequences were compared with the FV inoculum consensus, excluding variations that were already detected in the inoculum. To quantify mutational loads, the total, G-to-A and mA3-associated mutational frequencies relative to the consensus were divided by the total number of base pairs, G nucleotides and GG/GA dinucleotides analyzed, respectively.

### FV mutational analysis of newly formed reverse transcripts in target cells

To bias the detection of G-to-A mutations, we combined four approaches [11], [41]: (1) reverse transcripts were amplified following a single-round infection of *Mus dunni* cells, to enrich for potentially defective reverse transcripts; (2) a segment of *env* closest to the primer binding site was chosen, since this region may be present in single-stranded DNA form for longer duration and thereby more susceptible to deamination [67]; (3) *Taq* polymerase was utilized instead of *Pfu* polymerase, since *Pfu* polymerase activity is inhibited by deoxyuridines in DNA templates; and (4) a denaturation temperature of 88°C was used to enrich the detection of G-to-A hypermutated reverse transcripts, which should have a lower melting temperature. 7 dpi plasma samples (2.5 µl) from wild-type and B6 *mA3* ^−^ F_1_ mice were inoculated into *Mus dunni* cells in a 6-well plate containing 4 µg/ml polybrene. DNA was extracted after 2 days using the DNAeasy kit (Qiagen). PCR was performed with 10 ng DNA, 1× Sweet PCR mix (SA Biosciences), 2.5 mM dNTP, 1.25 pmol *env* primers FV.f and FV.r. Thermocycling conditions included a 95°C 15 min hot-start, followed by 30 cycles of denaturation at 88°C for 30 s, 55°C for 30 s and 72°C for 1.5 min. Amplicons were cloned using the TOPO-TA cloning kit (Invitrogen) and 4–8 clones were sequenced for each sample. Consensus from the FV inoculum and the corresponding plasma viral RNA sequences were used as reference for analysis of nucleotide substitutions using the HYPERMUT 2.0 software (hiv.lanl.gov).

### Quantification of early and late reverse transcripts

DNA samples as described above were subjected to absolute quantifications for early (R-U5) and late (R-gag) FV transcripts, as well as a housekeeping gene, beta-actin, using a Taqman assay (Applied Biosystems; Foster City, CA) in a CFX96 real-time system (Bio-Rad; Hercules, CA). The primers and probes are listed as follows. Early reverse transcripts: R-U5.fwd, CTCCGATAGACTGAGTCG, R-U5.rev, AGACCCTCCCAAGGAACA, R-U5.probe FAM-CCCGTGTATCCAATAAATCCTCTTGC-TAMRA. PCR cycling conditions were 95°C 10 min followed by 40 cycles of 95°C for 15 sec and 55.7°C for 45 sec. The expected size of PCR product is 94 bp. Late reverse transcripts: R-U5.fwd and R-Gag.rev, TTCGACATCCTTCCAGTGGT and R-Gag.probe, FAM-CTGCAGCATCGTTCTGTGT-TAMRA. The PCR conditions for R-Gag were 95°C for 10 min followed by 40 cycles of 95°C for 15 sec and 61°C for 2 min 30 sec. The expected size of the R-Gag amplicon is 670 bp. Mouse beta-actin: Actin.fwd, GGCACCACACCTTCTACAATG, Actin.rev, GGGGTGTTGAAGGTCTCAAAC, and Actin.probe FAM-TGTGGCCCCTGAGGAGCACCC-TAMRA. PCR conditions included a hot-start for 95°C 10 min followed by 40 cycles of 95°C for 15 sec and 60°C for 50 sec. Absolute copy numbers were interpolated from a best-fit standard curve against a DNA standard.

### Flow cytometry

Bone marrow and spleen cells (10^6^ cells) were stained with MAb 34, an IgG2b monoclonal antibody specific for FV Glyco-Gag [44] for 30 min, then co-stained with: Ter119-FITC (clone TER-119), CD3-Alexa700 (17A2), (BD Biosciences; San Diego, CA); CD11c-PE-Cy7 (N418), (eBioscience; San Diego, CA); CD19-PerCP-Cy5.5 (6D5) (Biolegend; San Diego, CA) and anti-mouse IgG2b-APC (Columbia Biosciences; Columbia, MD). Isotype controls and cells from uninfected mice were used for gating. Cells were processed in an LSR-II flow cytometer (BD Biosciences), collecting up to 250,000 events per sample. Datasets were analyzed using the Flowjo software (Treestar; Ashland, OR).

### Neutralizing antibody assay

Serial fourfold dilutions of heat-inactivated plasma were incubated with F-MuLV-N stock virus in the presence of guinea pig complement (Sigma). The antibody∶virus mixture was added into *M. dunni* cells and developed as in the plasma virus titrations [20]. The number of colonies were counted and compared to a no antibody control, which was set as 100%. Neutralization curves were constructed, and IC_50_ values were calculated based on a one-site sigmoidal fit using the Graphpad Prism software (Irvine, CA).

### Virion preparation

Virion antigens were prepared from culture supernatants obtained from *Mus dunni* cells infected with F-MuLV-N in T-175 flasks in the presence of 4 µg/ml polybrene (Sigma). Cellular debris was pelleted at 1800× *g* for 5 min at 4°C, then the supernatants were passed through a 0.22 µm filter. The filtered supernatants were ultracentrifuged at 25,000× *g* for 2 hr at 4°C in SW28 Ultra-Clear tubes (Beckman Coulter; Brea, CA), and virion pellets were resuspended in 0.5 ml Tris Buffered Saline (TBS) containing 1× protease inhibitor cocktail (Calbiochem; La Jolla, CA) per tube and allowed to dissociate overnight at 4°C. In some preparations, the virion pellets were resuspended in TBS with 1% Empigen-BB (Sigma), a zwitterionic detergent that is commonly used to solubilize viral particles and liberate envelope monomers [68], [69]. Virions were aliquoted and total protein concentrations were determined using a BCA protein assay (Pierce; Rockford, IL).

### Enzyme-linked immunosorbent assays (ELISAs)

ELISAs were performed at room temperature and 100 µl/well volumes unless otherwise indicated. Virions (200 ng per well) were coated into Immulon-4 HBX plates (Thermo Scientific Nunc; Rochester, NY) overnight at 4°C and blocked with SuperBlock (Pierce; Rockford, IL) for 2 hr. Serial 2-fold dilutions of plasma in Phosphate Buffered Saline (PBS) were added and incubated for 1 hr. After 6 washes with PBS with 0.05% Tween-20 (PBS-T), 1∶4000 biotinylated goat anti-mouse IgG (Southern Biotechnology; Birmingham, AL) was added and incubated for 1 hr. After 6 PBS-T washes, 1∶4000 streptavidin-conjugated horseradish peroxidase (Southern Biotechnology) was added and incubated for 30 min. Following 6 PBS-T washes, 100 µl of TMB substrate (BioFX Laboratories; Owings Mills, MD) was added per well and incubated in the dark for 15 min. The reaction was stopped with 0.3N sulfuric acid (Sigma), and absorbances were read at 450 nm in a Victor X5 plate reader (Perkin Elmer; Waltham, MA). The same procedures were followed for the IgM ELISAs, except that plasma samples were incubated overnight at 4°C, and 1∶10000 biotinylated goat anti-mouse IgM (Southern Biotechnology) was used. Endpoint titers were calculated by constructing one-site total best-fit curves using the Graphpad Prism software, and interpolating plasma concentrations that correspond to a cut-off value based on twice the mean absorbance background from wells with no plasma added. Samples from uninfected mice were also used as negative controls, and had absorbance values below the cut-off value (data not shown). Endpoint titers were expressed as log_2_ concentrations.

### Statistical analyses

Differences between means were analyzed using a two-tailed Student's *t* test. The association between G-to-A mutations with B6 *mA3* status was inferred by subjecting 2×2 contigency tables to a two-tailed Chi-square test. Differences with *p* values >0.05 were considered not statistically significant (N.S.).

## Supporting Information

Text S1 Supporting Figures S1 to S5 are presented with the corresponding legends.
(PDF)
