# Supplementary material for: Noninfectious Retrovirus Particles Drive the Apobec3/Rfv3 Dependent Neutralizing Antibody Response
Source: PLoS Pathog. 2011 Oct 6;7(10):e1002284. doi: 10.1371/journal.ppat.1002284 (PMC3188525; doi:10.1371/journal.ppat.1002284)
Supplement: Text S1 — Supporting Figures S1 to S5 are presented with the corresponding legends. (PDF) [file ppat.1002284.s001.pdf]

SUPPORTING INFORMATION

Figure S1. Rationale for F_1 transcomplementation to study *mA3/Rfv3*. (A) *Rfv3* is a dominant resistance gene found in B6 mice, but B6 mice are also resistant to splenomegaly (*Fv2*$^{r/r}$). In contrast, BALB/c mice are susceptible to splenomegaly (*Fv2*$^{s/s}$) and are also *Rfv3*$^{s/s}$. *Fv2* is a dominant susceptibility gene, such that hybrid (B6 × BALB/c)F_1 (*Fv2*$^{r/s}$) develop splenomegaly. However, since the *Rfv3* resistance gene is dominant, (B6 × BALB/c)F_1 eventually recover from infectious viremia. (B) To prove that *mA3* encodes *Rfv3*, B6 wild-type (*mA3*$^{+/+}$) or B6 *mA3*$^{-/-}$ mice were crossed to BALB/c mice to generate hybrid (B6 *mA3*$^{+/+}$ × BALB/c)F_1 and (B6 *mA3*$^{-/-}$ × BALB/c)F_1 mice. These congenic F_1 mice differ only in the presence or absence of the B6 *mA3* gene. Thus, the assembled cohorts in this study focus primarily on the functional consequences of B6 *mA3* expression. Note that the A.BY strain, like BALB/c, is *Fv2*$^{s/s}$ and *Rfv3*$^{s/s}$.

Figure S2. Noninfectious particle release during acute FV infection of (B6 × A.BY)F_1 mice. The (B6 × A.BY)F_1 background is more resistant than (B6 × BALB/c)F_1 mice due to stronger cellular immunity as governed by an *H-2*$^{b/d}$ haplotype. (A) Infectious viremia as measured by the *Mus dunni* assay was significantly higher in (B6 *mA3*$^{-/-}$ × A.BY)F_1 mice. (B) Plasma viral RNA loads between the two cohorts were not significantly different. (C) Virion infectivity, as measured by the ratio of $\log_{10}$ infectious titer and plasma viral load, was significantly higher in (B6 *mA3*$^{-/-}$ × A.BY)F_1 mice. (D) Normalized virion infectivity was computed with non-log transformed values, setting the average infectious titer per viral copy number of (B6 *mA3*$^{-/-}$ × A.BY)F_1 as 100%. Samples below the assay limit of detection (below the dotted lines in panel A) were excluded in this calculation. Solid lines correspond to mean values, and *p* values from a two-tailed Student's *t* test are shown. Error bars correspond to the standard error of the mean.

Figure S3. Sequence alignment of FV envelope segments from 7 dpi plasma viral RNA. Translated amino acid sequences were compared with the consensus FV stock inoculum sequence and disruptions in the open reading frame that led to a premature stop were evaluated. The impact of nucleotide mutations in specific codons on the amino acid sequence were highlighted in yellow. Out of 23 FV *env* sequences evaluated from each strain, (B6 $mA3^{+/+}$ × BALB/c)F_1 status was not associated with increased defective transcripts relative to (B6 $mA3^{-/-}$ × BALB/c)F_1 mice, consistent with a nonenzymatic mechanism of B6 *mA3* restriction of acute plasma virion infectivity.

Figure S4. IgM titers against native virions do not correlate with B6 *mA3* dependent NAb responses. Endpoint ELISAs were performed on individual plasma samples against native virions from (A) (B6 × BALB/c)F_1 and (B) (B6 × A.BY)F_1 mice. In both genetic backgrounds, the IgM response against FV was not significantly different in the presence or absence of B6 *mA3*. Values correspond to log_2-transformed plasma reciprocal dilutions that corresponded to a cut-off of 2× mean background absorbance. Differences in means were analyzed using a two-tailed Student's *t*-test.

Figure S5. B6 *mA3* dependent IgG against native virions in (B6 × A.BY)F_1 mice. Endpoint ELISAs were performed on individual plasma samples against (A) native virions and (B) detergent-lysed virions. Values correspond to log_2-transformed plasma reciprocal dilutions that corresponded to a cut-off based on 2× mean background absorbance. Differences in means were analyzed using a two-tailed Student's *t*-test.

Figure S1.

A

	**B6**	**BALB/c**	**(B6 × BALB/c) F_1**
Splenomegaly Induction	No<br>*Fv2*$^{r/r}$	Yes<br>*Fv2*$^{s/s}$	Yes<br>*Fv2*$^{r/s}$
Recovery from Viremia	Yes<br>*Rfv3*$^{r/r}$	No<br>*Rfv3*$^{s/s}$	Yes<br>*Rfv3*$^{r/s}$

B

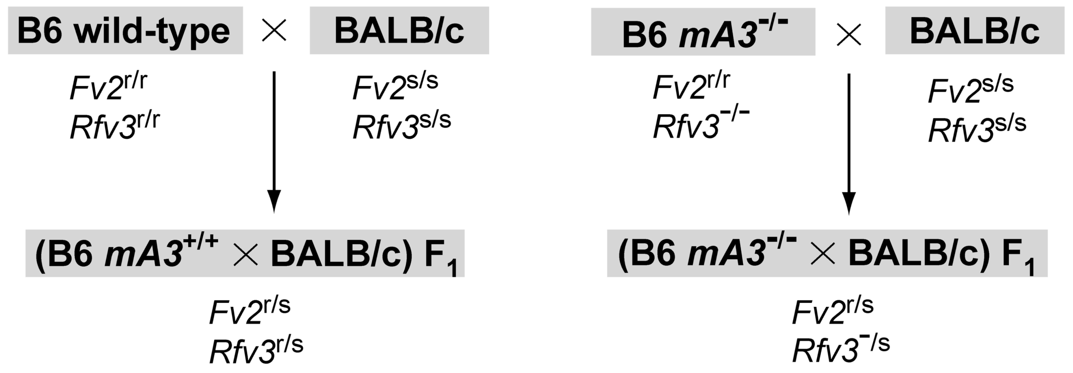

Figure S2.

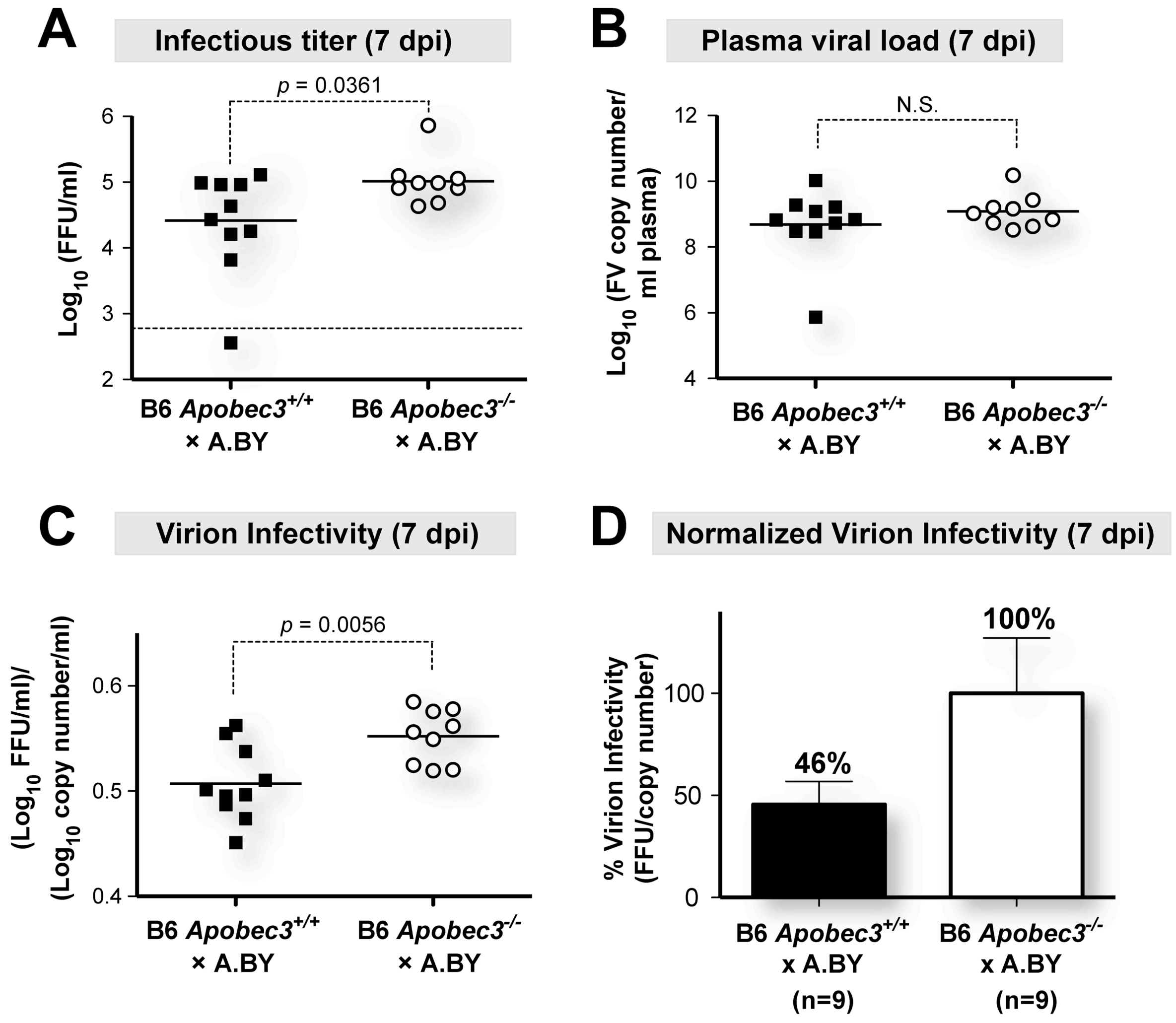

A
Infectious titer (7 dpi)
p = 0.0361
Log10 (FFU/ml)
6
5
4
3
2
B6 Apobec3+/+
× A.BY
B6 Apobec3-/-
× A.BY
B
Plasma viral load (7 dpi)
N.S.
Log10 (FV copy number/
ml plasma)
12
10
8
6
4
B6 Apobec3+/+
× A.BY
B6 Apobec3-/-
× A.BY
C
Virion Infectivity (7 dpi)
p = 0.0056
(Log10 FFU/ml)/
(Log10 copy number/ml)
0.6
0.5
0.4
B6 Apobec3+/+
× A.BY
B6 Apobec3-/-
× A.BY
D
Normalized Virion Infectivity (7 dpi)
% Virion Infectivity
(FFU/copy number)
100
50
0
46%
100%
B6 Apobec3+/+
x A.BY
(n=9)
B6 Apobec3-/-
x A.BY
(n=9)

Figure S3.

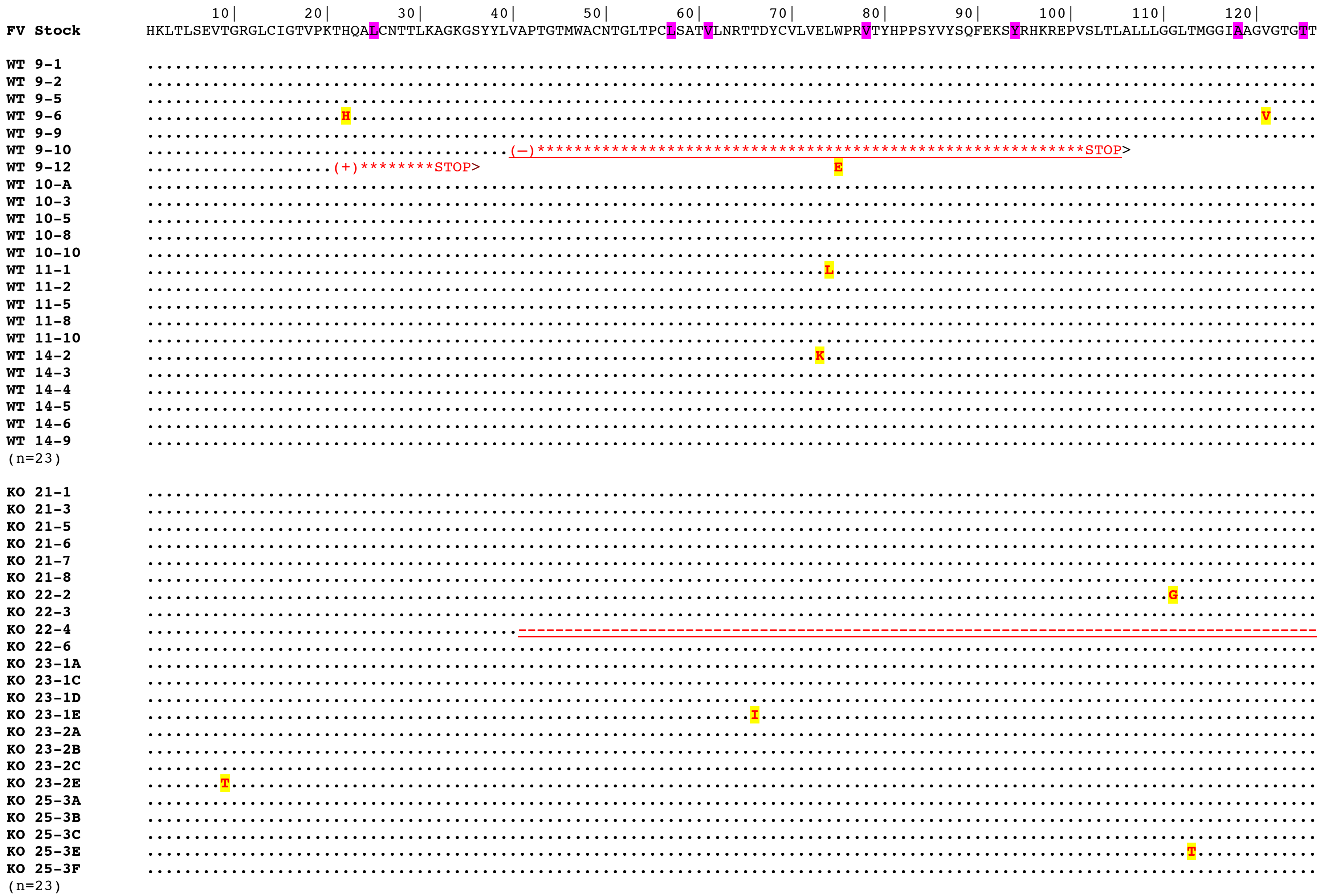
10 20 30 40 50 60 70 80 90 100 110 120
FV Stock HKLTLSEVTGRGLCIGTVPKTHQALCNTTLKAGKGSYYLVAPTGTMWACNTGLTPCLSATVLNRTTDYCVLVELWPRVTYHPPSYVYSQFEKSYRHKREPVSLTLALLLGGLTMGGIAAGVGTGTT
WT 9-1
WT 9-2
WT 9-5
WT 9-6 H V
WT 9-9
WT 9-10 (–)*************STOP>
WT 9-12 (+)********STOP> E
WT 10-A
WT 10-3
WT 10-5
WT 10-8
WT 10-10
WT 11-1 L
WT 11-2
WT 11-5
WT 11-8
WT 11-10
WT 14-2 K
WT 14-3
WT 14-4
WT 14-5
WT 14-6
WT 14-9
(n=23)
KO 21-1
KO 21-3
KO 21-5
KO 21-6
KO 21-7
KO 21-8
KO 22-2 G
KO 22-3
KO 22-4
KO 22-6
KO 23-1A
KO 23-1C
KO 23-1D
KO 23-1E I
KO 23-2A
KO 23-2B
KO 23-2C
KO 23-2E T
KO 25-3A
KO 25-3B
KO 25-3C
KO 25-3E T
KO 25-3F
(n=23)

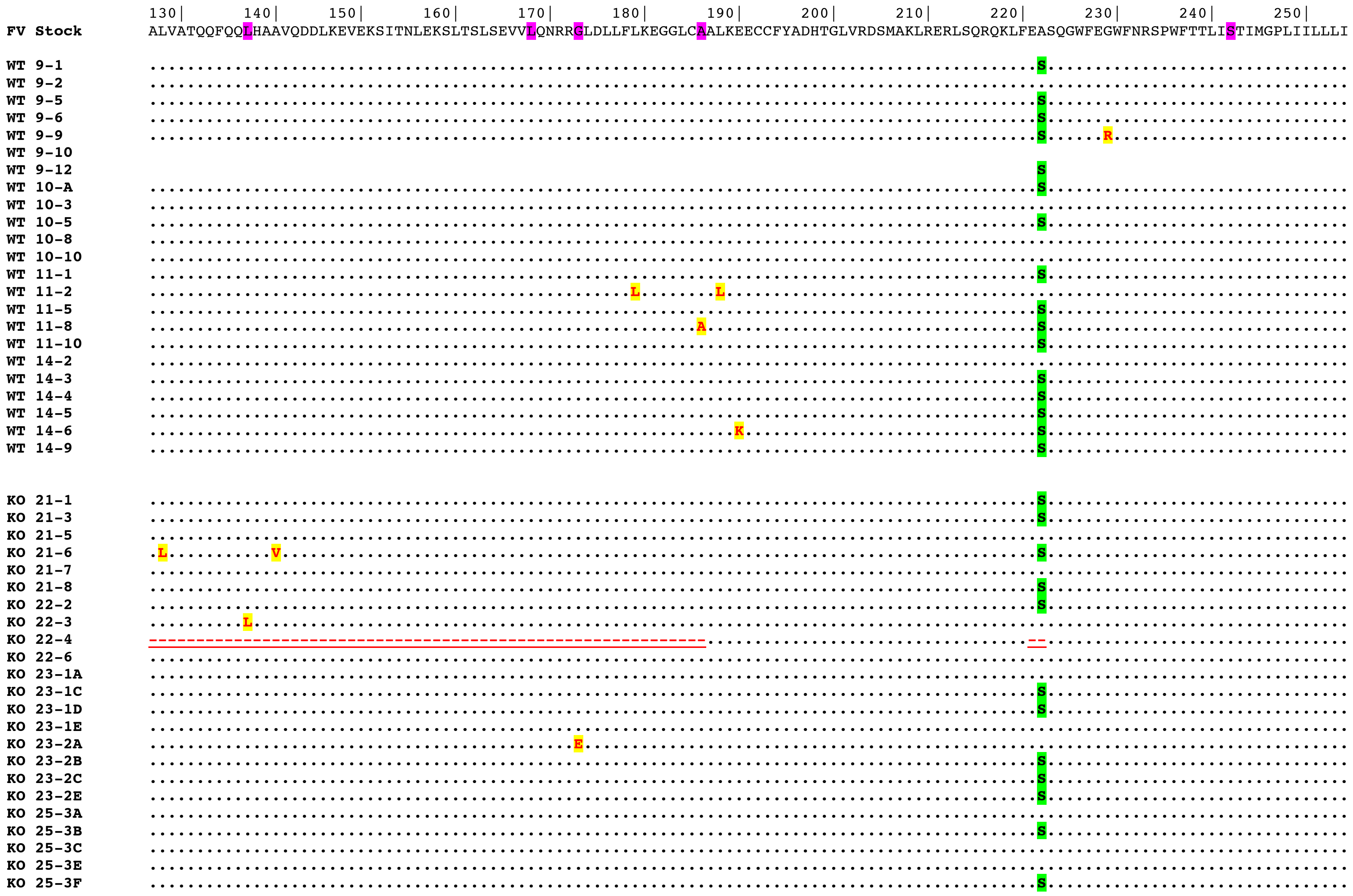

130 140 150 160 170 180 190 200 210 220 230 240 250
FV Stock
ALVATQQFQQLHAAVQDDLKEVEKSITNLEKSLTSLSEVVLQNRRGLDLLFLKEGGLCAALKEECCFYADHTGLVRDSMAKLRERLSQRQKLFEASQGWFEGWFNRSPWFTTLISTIMGPLIILLLI
WT 9-1
WT 9-2
WT 9-5
WT 9-6
WT 9-9
WT 9-10
WT 9-12
WT 10-A
WT 10-3
WT 10-5
WT 10-8
WT 10-10
WT 11-1
WT 11-2
WT 11-5
WT 11-8
WT 11-10
WT 14-2
WT 14-3
WT 14-4
WT 14-5
WT 14-6
WT 14-9
KO 21-1
KO 21-3
KO 21-5
KO 21-6
KO 21-7
KO 21-8
KO 22-2
KO 22-3
KO 22-4
KO 22-6
KO 23-1A
KO 23-1C
KO 23-1D
KO 23-1E
KO 23-2A
KO 23-2B
KO 23-2C
KO 23-2E
KO 25-3A
KO 25-3B
KO 25-3C
KO 25-3E
KO 25-3F

```
                       260|      270|      280|
FV Stock           LLFGPCILNRLVQFVKDRISVVQALVLT

WT 9-1             ............................
WT 9-2             .......................T....
WT 9-5             ............................
WT 9-6             ............................
WT 9-9             ............................
WT 9-10
WT 9-12
WT 10-A            ............................
WT 10-3            ............................
WT 10-5            ............................
WT 10-8            ............................
WT 10-10           ............................
WT 11-1            ............................
WT 11-2            .....................V......
WT 11-5            ............................
WT 11-8            ............................
WT 11-10           ............................
WT 14-2            ............................
WT 14-3            ............................
WT 14-4            ............................
WT 14-5            ............................
WT 14-6            ............................
WT 14-9            ............................


KO 21-1            ............................
KO 21-3            ............................
KO 21-5            ............................
KO 21-6            ............................
KO 21-7            ............................
KO 21-8            ............................
KO 22-2            ............................
KO 22-3            ............................
KO 22-4            ............................
KO 22-6            ............................
KO 23-1A           ......L.....................
KO 23-1C           ............................
KO 23-1D           ............................
KO 23-1E           ............................
KO 23-2A           ............................
KO 23-2B           ............................
KO 23-2C           ........................L...
KO 23-2E           ............................
KO 25-3A           ............................
KO 25-3B           ............................
KO 25-3C           L...........................
KO 25-3E           ...............K............
KO 25-3F           ............................
```

Key:

Base change found in FV inoculum

Nucleotide change found in plasma.

Mixed population

Nucleotide Addition(+)/deletion(-) that cause frameshift stop codons: (+)*****STOP

Large deletion: ----------

Figure S4.

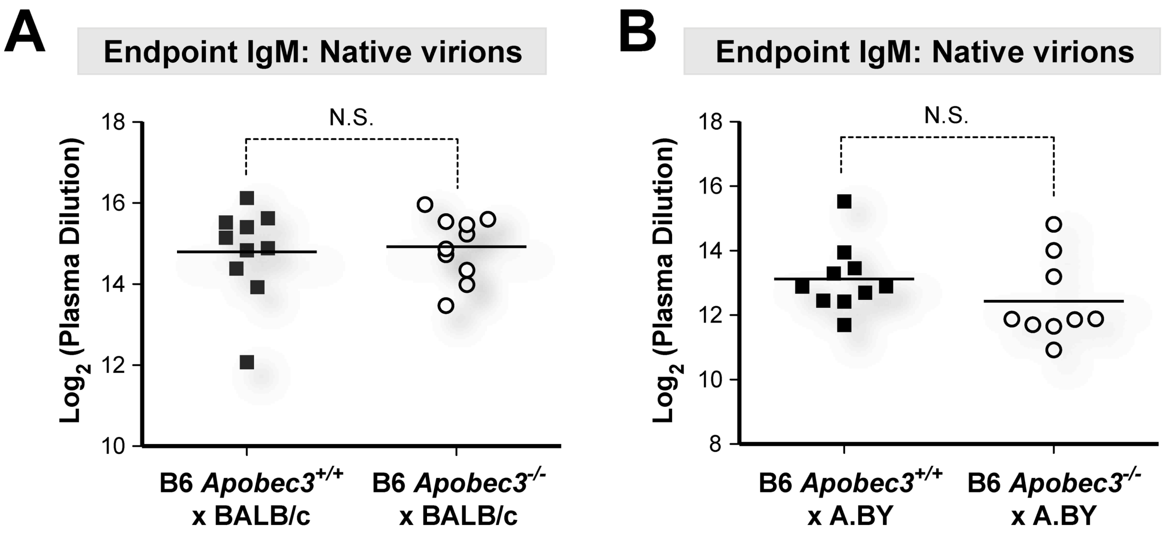
A
Endpoint IgM: Native virions
N.S.
Log2 (Plasma Dilution)
18
16
14
12
10
B6 Apobec3+/+ x BALB/c
B6 Apobec3-/- x BALB/c
B
Endpoint IgM: Native virions
N.S.
Log2 (Plasma Dilution)
18
16
14
12
10
8
B6 Apobec3+/+ x A.BY
B6 Apobec3-/- x A.BY

Figure S5.

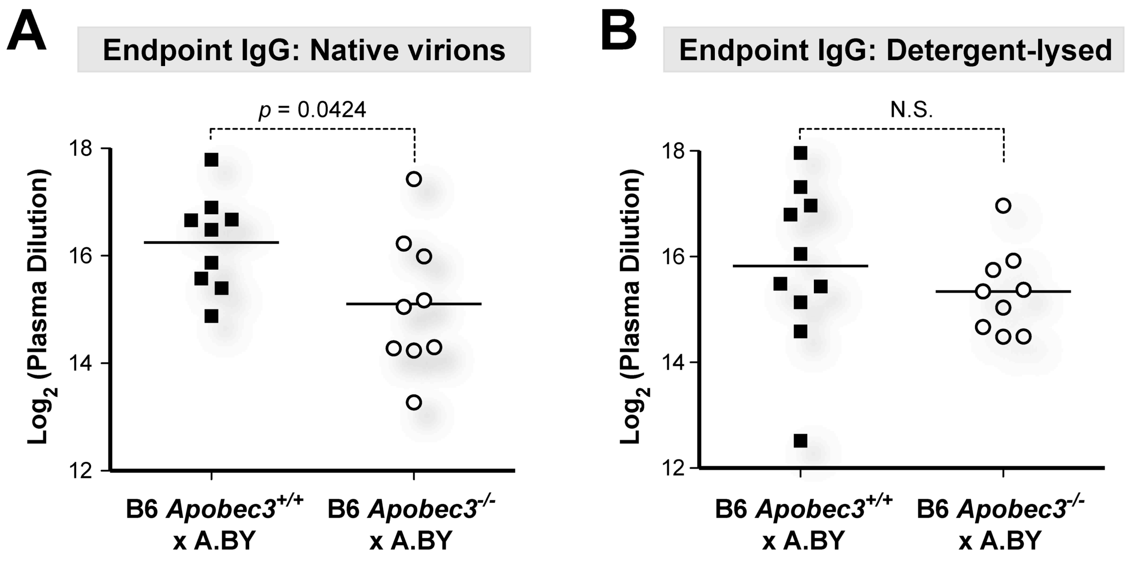
A
Endpoint IgG: Native virions
p = 0.0424
Log2 (Plasma Dilution)
18
16
14
12
B6 Apobec3+/+ x A.BY
B6 Apobec3-/- x A.BY
B
Endpoint IgG: Detergent-lysed
N.S.
Log2 (Plasma Dilution)
18
16
14
12
B6 Apobec3+/+ x A.BY
B6 Apobec3-/- x A.BY
